# The Ontogeny of Monocyte Subsets

**DOI:** 10.3389/fimmu.2019.01642

**Published:** 2019-07-17

**Authors:** Anja A. Wolf, Alberto Yáñez, Pijus K. Barman, Helen S. Goodridge

**Affiliations:** ^1^Board of Governors Regenerative Medicine Institute, Cedars-Sinai Medical Center, Los Angeles, CA, United States; ^2^Research Division of Immunology, Department of Biomedical Sciences, Cedars-Sinai Medical Center, Los Angeles, CA, United States; ^3^Departament de Microbiologia i Ecologia, Universitat de València, Burjassot, Spain; ^4^Estructura de Recerca Interdisciplinar en Biotecnologia i Biomedicina, Universitat de València, Burjassot, Spain

**Keywords:** monocyte subsets, monocyte progenitors, monocyte ontogeny, monopoiesis, bone marrow

## Abstract

Classical and non-classical monocytes, and the macrophages and monocyte-derived dendritic cells they produce, play key roles in host defense against pathogens, immune regulation, tissue repair and many other processes throughout the body. Recent studies have revealed previously unappreciated heterogeneity among monocytes that may explain this functional diversity, but our understanding of mechanisms controlling the functional programming of distinct monocyte subsets remains incomplete. Resolving monocyte heterogeneity and understanding how their functional identity is determined holds great promise for therapeutic immune modulation. In this review, we examine how monocyte origins and developmental influences shape the phenotypic and functional characteristics of monocyte subsets during homeostasis and in the context of infection, inflammation, and cancer. We consider how extrinsic signals and transcriptional regulators impact monocyte production and functional programming, as well as the influence of epigenetic and metabolic mechanisms. We also examine the evidence that functionally distinct monocyte subsets are produced via different developmental pathways during homeostasis and that inflammatory stimuli differentially target progenitors during an emergency response. We highlight the need for a more comprehensive understanding of the relationship between monocyte ontogeny and heterogeneity, including multiparametric single-cell profiling and functional analyses. Studies defining mechanisms of monocyte subset production and maintenance of unique monocyte identities have the potential to facilitate the design of therapeutic interventions to target specific monocyte subsets in a variety of disease contexts, including infectious and inflammatory diseases, cancer, and aging.

## Introduction

Monocytes are innate immune cells of the myeloid lineage that are produced throughout life and play diverse roles all over the body, including in tissue development and homeostasis, host defense, initiation and resolution of inflammation, and tissue repair. They are produced during homeostasis by hematopoietic stem and progenitor cells (HSPCs) in the bone marrow (steady-state monopoiesis), and their production is enhanced during “emergency monopoiesis,” which occurs in diverse circumstances including in response to infectious and inflammatory stimuli, in the presence of tumors, and during chronic psychosocial stress (1–5). In addition to quantitative changes, there are also qualitative changes with the production of functionally distinct monocytes and monocyte-derived cells in response to stress.

Monocytes initially arise in the fetal liver from late yolk sac-derived erythromyeloid progenitors during the transient-definitive wave of hematopoiesis, from around embryonic day 8.5 (E8.5) in mice [reviewed in (6)]. At E10.5, immature hematopoietic stem cells (HSCs), which arise from the aorta-gonad-mesonephros (AGM) region of the embryo, colonize, and establish definitive hematopoiesis in the fetal liver, which serves as the major hematopoietic organ for the developing immune system. HSCs subsequently seed the bone marrow but are not fully functional until several days after birth, so liver (and spleen) HSCs continue to supply monocytes during the peri-natal period until the establishment of nascent adult-like HSCs.

**Figure 1:**
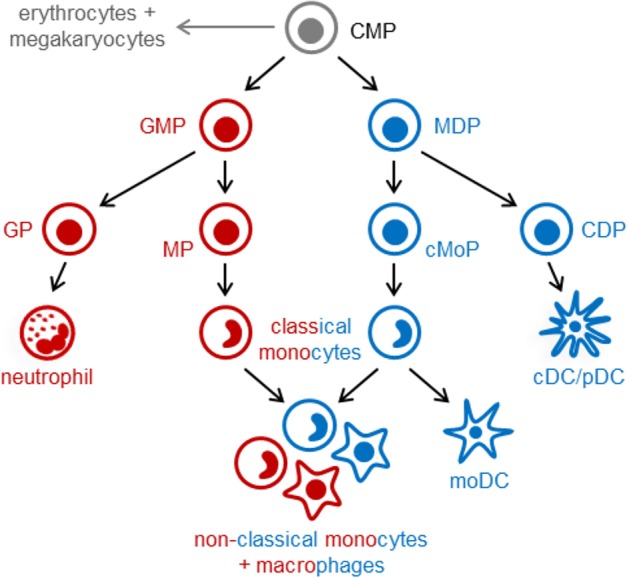
Pathways of myeloid cell differentiation. In the steady-state, distinct mouse monocyte subsets arise independently from common myeloid progenitors (CMPs; LKS^−^ CD34^+^ FcγR^lo^ Flt3^+^ CD115^lo^ cells) via granulocyte-monocyte progenitors (GMPs; LKS^−^ CD34^+^ FcγR^hi^ Ly6C^−^) and monocyte-DC progenitors (MDPs; LKS^−^ CD34^+^ FcγR^lo^ Flt3^+^ CD115^hi^). GMPs also produce neutrophils (via granulocyte progenitors, GPs), and MDPs yield cDCs and pDCs (via common DC progenitors, CDPs). Functionally distinct subsets of classical monocytes (Ly6C^hi^ in mice) are produced by both GMPs and MDPs. Non-classical (Ly6C^−^) monocytes and macrophages, also arise via both pathways and may exhibit functional differences. In contrast, monocyte-derived DCs (moDCs, which are ontogenetically and functionally distinct from cDCs and pDCs) arise exclusively from MDP-derived monocytes, and GMPs produce a neutrophil-like subset of classical monocytes. Monocyte-committed progenitors arising from GMPs (known as MPs) and MDPs (known as cMoPs) are both found in the LKS^−^ CD34^+^ FcγR^hi^ Ly6C^+^ CD115^hi^ fraction of mouse bone marrow; it is not currently possible to separate them using surface markers, but MPs and cMoPs are revealed as distinct cell clusters by single-cell RNA sequencing.

In the post-natal bone marrow, monocytes are produced by HSCs via progenitors with progressively restricted lineage potential that ultimately commit to monocyte production. In both mice and humans, monocytes arise from multilineage common myeloid progenitors (CMPs), which also produce neutrophils, dendritic cells (DCs), erythrocytes and megakaryocytes (7, 8). Monocytes arise via two independent pathways in mice (Figure 1) and probably also in humans: granulocyte-monocyte progenitors (GMPs) produce monocytes and neutrophils, and monocyte-DC progenitors (MDPs) yield monocytes as well as conventional and plasmacytoid DCs (cDCs and pDCs) (7, 9–11). Monocyte-committed progenitors—GMP-derived MPs and MDP-derived cMoPs, which are discussed in more detail below—have been isolated in both mice and humans (11–14). The adult spleen also contains a reservoir of monocytes that can be rapidly recruited in response to injury or inflammation (15). Extramedullary monopoiesis has also been reported in the adult mouse spleen under inflammatory conditions, including in the presence of tumors, in models of psychosocial stress, and upon aging (3, 16–18).

Recent studies have revealed the diverse origins of macrophages resident in different tissues [reviewed in (6, 19)]. Microglia arise exclusively from yolk sac progenitors, independent of HSCs and monocytes. Other tissue macrophages are monocyte-derived, but with diverse temporal origins. Langerhans cells, alveolar macrophages, and Kupffer cells, for instance, initially originate from yolk sac progenitors, but are subsequently replaced by fetal liver-derived monocytes. Macrophages in the heart, pancreas, gut and dermis, in contrast, are originally derived from fetal liver monocytes, but significantly or almost entirely replaced by bone marrow-derived monocytes after birth, or later in life, in a tissue-specific manner.

Two major types of monocytes have been extensively characterized in both mice and humans: classical (Ly6C^hi^ CD43^−^ monocytes in mice, which correspond to CD14^+^ CD16^−^ monocytes in humans) and non-classical (Ly6C^lo^ CD43^+^ monocytes in mice, which correspond to CD14^lo^ CD16^+^ monocytes in humans) (20–23). In the steady-state, reserves of classical monocytes are maintained in the bone marrow and other extramedullary sites, such as the spleen, where they are available for immediate deployment to infected or injured tissues and can give rise to macrophages or monocyte-derived dendritic cells (moDCs) with diverse roles in controlling infection, limiting inflammatory damage, and initiating tissue repair. Non-classical monocytes, on the other hand, are recruited to non-inflamed tissues in a CX3CR1-dependent manner, and are characterized by their ability to patrol the resting vasculature, remove cell debris, and repair the endothelium during homeostasis (21–25).

Non-classical monocytes are less proliferative than classical monocytes, but they remain in the circulation longer (25, 26). Most evidence indicates that they arise from classical monocytes in both mice and humans (23–26). Intermediate monocytes (Ly6C^int^ CD43^+^ monocytes in mice and CD14^+^ CD16^+^ monocytes in humans) have also been characterized (23, 26–30). They possess many of the inflammatory characteristics of classical monocytes, but express similar levels of CX3CR1 to non-classical monocytes, although they do not actively patrol the vasculature.

It is, however, becoming increasingly clear that monocytes are much more heterogeneous than previously appreciated. In this review, we highlight recent insights into the production and programming of monocytes with distinct functional attributes during homeostasis and in the context of infection, inflammation and cancer. We discuss how monocyte origins influence their function by considering the developmental pathways of monocyte production and reviewing how monocyte function is programmed during differentiation and influenced by signals that instruct or promote monopoiesis.

## Monocyte Heterogeneity

Multiparametric single-cell studies using flow cytometry, mass cytometry and single-cell RNA sequencing have recently revealed further heterogeneity among mouse and human monocytes, and combined with functional studies, have provided insight to support the identification of monocyte subsets. Some subsets are distinct stages of a linear differentiation pathway, whereas others represent functionally distinct monocytes, including new subsets that arise under emergency conditions. In the context of infection and inflammation, for example, elevated monocyte numbers may reflect amplification of steady-state subsets and/or the appearance of new populations with the ability to promote inflammatory responses, initiate tissue healing, or induce fibrosis.

The CXCR4^+^ subset of Ly6C^hi^ monocytes in mouse bone marrow is a transient population of pre-monocytes that lose CXCR4 expression as they mature, which facilitates their exit from the bone marrow (31). Similarly, TREML4^−^ Ly6C^hi^ monocytes can produce Zbtb46^+^ moDCs capable of cross-priming CD8^+^ T cells, whereas it appears that TREML4^+^ Ly6C^hi^ monocytes are intermediate monocytes that have lost the potential to produce moDCs, but can still give rise to Ly6C^lo^ monocytes, which are also TREML4^+^ (32). A recent single-cell RNA sequencing study also revealed heterogeneity among human intermediate monocytes (29), which may at least in part reflect different stages of classical to non-classical conversion.

Surface expression of 6-sulfo LacNAc (slan), a carbohydrate modification of P-selectin glycoprotein 1 (PSGL1), has been reported to distinguish intermediate (slan^−^), and non-classical (slan^+^) human monocytes (33), although a recent mass cytometry study revealed a subset of slan^−^ non-classical monocytes (30). The latter study reported 8 monocyte subsets in peripheral blood from healthy human subjects, including CD61^+^ and CD9^+^ subsets of non-classical monocytes (30). The CD9^+^ subset was also detected in mice and likely reflects platelet binding to these monocytes.

Functionally distinct moDCs—CD103^+^ DCs produced by Ly6C^hi^ CCR2^hi^ monocytes, and CD11b^hi^ DCs derived from Ly6C^lo^ CCR2^lo^ monocytes—have previously been reported in the steady-state lung (34). Moreover, a recent study described two distinct populations of Ly6C^hi^ monocyte-derived macrophages resident in multiple mouse and human tissues: antigen-presenting Lyve1^lo^ MHCII^hi^ CX3CR1^hi^ macrophages located adjacent to nerve bundles and fibers, and Lyve1^hi^ MHCII^lo^ CX3CR1^lo^ macrophages, which reside near blood vessels and are functionally optimized for tissue repair (35).

Distinct mouse monocyte subsets detectable in the steady-state have also been reported to give rise to inflammatory macrophages and monocyte-derived DCs (36). Expression of MHCII and CD209a (one of the eight mouse homologs of DC-SIGN) defines a subset of Ly6C^hi^ monocytes present in small numbers in the steady-state (~5% Ly6C^hi^ monocytes in the bone marrow) that are capable of differentiating into moDCs under inflammatory conditions, whereas Ly6C^hi^ monocytes lacking CD209a and MHCII expression (~90% of Ly6C^hi^ monocytes) produce iNOS^+^ inflammatory macrophages.

Single-cell RNA sequencing has also revealed a population of steady-state Ly6C^hi^ monocytes (~15%) with neutrophil-like properties, including strong expression of granule proteins (11). Monocytes with a neutrophil-like gene signature have also been reported in mouse and human lung tumors and peripheral blood (37). In humans, these monocytes were classical (CD14^+^), whereas the mouse equivalents, which were also present in tumor-free lung tissue, included both classical and non-classical (Ly6C^hi^ and Ly6C^lo^) monocytes. Moreover, fibrosis-promoting Ceacam1^+^ Msr1^+^ non-classical monocytes with granulocytic properties (named segregated-nucleus-containing atypical monocytes, SatMs) are not detected in the steady-state, but appear *de novo* following bleomycin administration (38). It is currently unclear whether or how these neutrophil-like subsets are ontogenetically or functionally related to one another.

**Figure 2:**
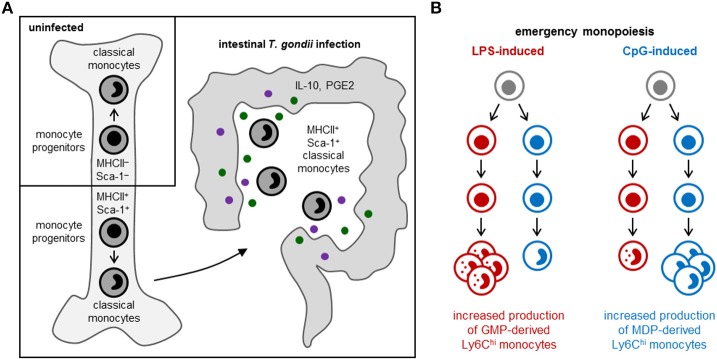
Emergency monopoiesis. Under emergency or stress conditions, functionally distinct monocyte subsets may arise in the bone marrow or spleen, and production of monocyte (and other myeloid cell) subsets may be selectively enhanced. **(A)** In response to intestinal *T. gondii* infection in mice, MHCII^+^ Sca-1^+^ CX3CR1^−^ Ly6C^hi^ monocytes are produced by monocyte-committed progenitors that, unlike their steady-state counterparts, also express MHCII and Sca-1 (40). **(B)** LPS and CpG promote monocyte production by murine GMPs and MDPs, respectively; LPS also stimulates neutrophil production by GMPs, whereas CpG enhances cDC production by MDPs (11).

Some monocyte subsets appear to contribute to tissue damage, whereas others promote tissue repair. Patients with coronary artery disease, for instance, have elevated numbers of slan^+^ CXCR6^+^ non-classical monocytes, which correlate with disease severity (30), and asthma severity in humans has been reported to correlate with elevated numbers of circulating TGF-β1-producing classical monocytes, which differentiate into fibrocytes instead of macrophage-like cells (39). In contrast, MHCII^+^ Sca-1^+^ CX3CR1^−^ Ly6C^hi^ monocytes, which are thought to limit immunopathology via production of prostaglandin E2 (PGE2) and IL-10, arise in the bone marrow following acute gastrointestinal infection with *Toxoplasma gondii* [(40); Figure 2A], and immunoregulatory Ym1^+^ Ly6C^hi^ monocytes have also been observed during the recovery phase of tissue injury (41).

Monocyte subsets that promote anti-tumor immunity, or conversely, support tumor growth have also been reported [reviewed in (5)]. For instance, tumor antigen-presenting CD103^+^ Ly6C^+^ monocytes have been reported to be required for efficient cross-presentation of tumor antigens and responsiveness to immunotherapy and immunogenic chemotherapy (42). In contrast, a subset of tumor-infiltrating pro-angiogenic Tie2^+^ non-classical monocytes has been described in tumors and the circulation of tumor-bearing mice and cancer patients (as well as healthy controls) (43, 44). Immunosuppressive classical monocytes, often termed monocytic myeloid-derived suppressor cells (M-MDSCs), are also prevalent in tumor-bearing mice and humans, as well as in inflammatory contexts such as sepsis and autoimmunity [reviewed in (4)]. They are characterized by their ability to suppress cytotoxic T cell and NK cell activation, promote anti-inflammatory and immunoregulatory responses (IL-10, regulatory T cells etc.), and support tumor progression and metastasis. However, in these contexts, it is unclear whether all classical monocytes exhibit these properties or just a fraction of them, because T cell suppression is evaluated using bulk populations of monocytic cells. A single-cell RNA sequencing study recently revealed 3 monocyte transcriptional states (both classical and non-classical, including the neutrophil-like monocytes mentioned above), as well as several macrophages and moDC subsets, in human and mouse lung tumors (37), although the functional significance of these subsets remains to be determined.

## Control of Monocyte Subset Production and Functional Programming

Signals from the microenvironment can influence monocyte gene expression and function in a tissue-specific manner, but the presence of multiple distinct subsets of monocytes or monocyte-derived cells in the same tissue indicates that they may have independent origins. We will consider how signals sensed by HSPCs, such as cytokines and microbial components, shape the repertoire of monocytes produced, both in the steady-state and under emergency conditions. This may occur via epigenetic and metabolic programming of the differentiating cells, which is discussed in this section, and/or via selective promotion of specific differentiation pathways that yield distinct monocyte subsets, which is considered in the next section.

Cytokines released by hematopoietic or non-hematopoietic cells in the bone marrow niche, or originating from outside the bone marrow via the circulation, can influence monocyte production and functional programming upon detection by progenitors. For instance, production of regulatory MHCII^+^ Sca-1^+^ CX3CR1^−^ Ly6C^hi^ monocytes in response to *T. gondii* infection is instructed by IFN-γ produced by NK cells in the bone marrow, and elevated expression of MHCII and Sca-1 is already evident at the monocyte progenitor stage [(40); Figure 2A]. Sca-1 upregulation by myeloid progenitors is also induced by other inflammatory stimuli, including type I interferons and TNF-α [reviewed in (1)]. HSPCs also express a variety of pattern recognition receptors, allowing them to directly sense microbes [reviewed in (45)]. Exposure of endogenous or purified HSPCs to whole microbes (bacteria, viruses and fungi) or microbial components induces them to produce monocytes and other myeloid cells, but can also program the function of the macrophages they produce. For instance, HSPCs exposed to TLR2 agonists differentiate into macrophages that are less inflammatory (produce lower levels of inflammatory cytokines and reactive oxygen species) than those derived from unexposed HSPCs (46).

Progenitor programming is also thought to underlie observations of innate immune memory. Although cells of the innate immune system do not possess the antigen-specific memory of T and B cells, epigenetic and metabolic changes induced by microbial stimuli can alter their responses to subsequent stimulation. Lipopolysaccharide, for example, can tolerize macrophage cytokine responses and prime microbicidal responses via selective chromatin remodeling (47). Similarly, detection of fungal β-glucans or the Bacillus Calmette-Guérin (BCG) vaccine trains monocytes and macrophages to enhance their responsiveness to secondary stimulation by inducing changes in histone modifications and a metabolic shift toward glycolysis (48, 49). Innate immune memory mechanisms are thought to last weeks, months or possibly even years, and may contribute to protection against subsequent infections. Recent studies have also demonstrated epigenetic and metabolic changes in HSPCs, which may contribute to the persistence of such effects. *In vivo* BCG administration induced myeloid progenitor expansion and sustained production of macrophages epigenetically trained to more effectively kill *Mycobacterium tuberculosis* (50). β-glucan injection similarly promoted myelopoiesis and induced metabolic alterations in progenitors, consistent with those observed in β-glucan-trained mature monocytes and macrophages (51).

Beyond microbial infection, it is likely that other stimuli also impact the functional programming of monocytes during differentiation, whether or not they concomitantly boost monocyte numbers. For instance, PGE2 induced by UV skin irradiation causes epigenetic and metabolic changes in bone marrow progenitors, and the monocytes, macrophages and DCs they produce have a restricted capacity to migrate in response to chemoattractants and inflammatory mediators (52–55). Immunosuppressive monocytes produced in the context of tumors may also acquire their suppressive properties during differentiation, because monocytic cells isolated from the circulation, spleen and bone marrow of cancer patients and tumor-bearing mice have been reported to inhibit T cell activation and promote tumor growth (4, 5, 56). Tumor-derived factors are therefore thought to instruct the programming of pro-tumor monocytes during differentiation, in addition to their local effects in the tumor itself.

The spleen has been shown to be a key source of monocytes and neutrophils recruited to tumors and may be an important site for monocyte functional programming. In murine lung cancer models, extramedullary myelopoiesis was detected in the spleen (16) and splenectomy reduced tumor progression (16, 56). The underlying mechanisms in these splenectomy models appear to vary with tumor type, but include reduced recruitment of monocytes to the tumors, fewer immunosuppressive monocytes, and more anti-tumor macrophages. Human patients with invasive pancreatic or colon cancer have also been reported to have more splenic myeloid progenitors and monocytes than individuals without invasive cancer (16). Myeloid progenitor recruitment from the bone marrow to the spleen has also been reported in a mouse model of psychosocial stress, in which splenic monopoiesis provides monocytes that traffic to the brain and induce anxiety-like behavior (3).

## Distinct Pathways of Monopoiesis

Distinct monocyte subsets may also arise via independent differentiation pathways. A study from our lab recently revealed the existence of two independent pathways of monopoiesis in the steady-state, which yield functionally distinct monocyte subsets (11). We demonstrated that neutrophil-like Ly6C^hi^ monocytes arise from GMPs (which also produce neutrophils), whereas MHCII^+^ CD209a^+^ Ly6C^hi^ monocytes capable of producing moDCs are derived from MDPs (which also produce cDCs and pDCs) (Figure 1). Other studies applying lineage trajectory analyses of single-cell RNA sequencing datasets similarly predicted the existence of two pathways of monocyte differentiation (57, 58). Macrophages produced via the two monocyte lineages are also probably functionally distinct. Indeed, we observed higher CD86 expression by CD11c^+^ MHCII^−/lo^ macrophages in GM-CSF cultures of MDP-derived monocytes compared to the same cell fraction in GMP-derived monocyte cultures (11). We also demonstrated that LPS and CpG differentially target the GMP and MDP pathways, respectively, to boost monocyte production (11) (Figure 2B), although it is currently unclear whether this effect is direct (due to TLR-mediated detection by the progenitors themselves) or indirect.

While the GMP vs. MDP origins of most monocyte subsets are as yet unknown, some emerging studies provide additional evidence of independent pathways for the production of distinct monocyte subsets. Immunoregulatory Ym1^+^ Ly6C^hi^ monocytes, for instance, are reportedly GMP-derived and not MDP-derived (41). New differentiation pathways may also exist under emergency conditions. In support of this possibility, SatMs arose from a subset of FcεRI^+^ GMPs independently of steady-state monocyte-committed progenitors in fibrosis models (38). Monocyte programming that is already evident at the progenitor level, as seen in the context of *T. gondii* infection (40), may specifically affect one pathway or have similar effects in both.

## Transcriptional Regulators of Monocyte Subset Production and Functional Programming

Several transcription factors have been implicated in steady-state and emergency monopoiesis, including some that govern the production of specific monocyte subsets. IRF8 is a key regulator of monocyte differentiation [reviewed in (59)]. It binds with PU.1 to promoters and enhancers to induce the monocyte lineage gene program. IRF8 is dispensable for monocyte lineage specification, but required for the production of steady-state monocytes by monocyte-committed progenitors, as evidenced in IRF8-deficient mice by the accumulation of monocyte-committed progenitors and monoblasts that fail to differentiate into mature monocytes (13). IRF8 induces the expression of monocyte genes both directly and indirectly via induction of the transcription factor Klf4 (60). IRF8 induces the formation of enhancers to direct the expression of monocyte genes (61), and also interacts with the transcription factor c/EBPα to inhibit the granulocyte program (62).

Zeb2 and GATA2 have also been implicated in monocyte differentiation. Zeb2 deletion results in depletion of Ly6C^hi^ monocytes in the bone marrow (63, 64), and GATA2 mutations have been identified in patients with monocyte deficiencies (65, 66). miR146a differentially regulates classical and non-classical monocytes, targeting transcripts for the non-canonical NF-κB family member RelB to restrict classical monocyte expansion during inflammatory challenge without affecting non-classical monocytes (67). We observed high expression of Gfi1, which is important for granulopoiesis, in GMP-derived neutrophil-like Ly6C^hi^ monocytes (11), but its role in the functional programming of these monocytes, including their expression of granule proteins, remains to be determined.

The conversion of Ly6C^hi^ monocytes to Ly6C^−^ monocytes is dependent on the transcription factor c/EBPβ, which is required for the survival of Ly6C^−^ monocytes and maintenance of CD115 expression, at least in part via induction of NR4A1 (Nur77) (24, 28, 68). c/EBPβ also regulates the production of SatM monocytes associated with the development of fibrosis (38), has been implicated in the production of M-MDSCs (69), and defines the enhancer landscape of moDCs (70). Monocyte differentiation into cross-priming moDCs also requires IRF4, but is BATF3-independent (32). In contrast, p53-drived BATF3 upregulation is reportedly required for differentiation of tumor antigen-presenting CD103^+^ Ly6C^+^ monocytes (42).

## Implications and Future Opportunities

A growing body of evidence therefore supports the notion that heterogeneity among monocytes in part reflects their origins. A major outstanding question is whether developmental influences have a prolonged impact on the functional programming of monocytes and their derivatives, or whether they are largely overridden by subsequent exposure to other stimuli (cytokines, microbes, tumors etc.) after monocytes leave the bone marrow. This applies in the context of both steady-state and emergency monopoiesis, including in relation to innate immune progenitor memory effects. The role of the splenic microenvironment is also of particular interest under emergency conditions when extramedullary myelopoiesis is often observed.

In terms of ontogeny, it will be important to define which monocyte subsets derive from GMPs vs. MDPs (or via other, as yet undescribed, pathways). For instance, it is unclear whether immunoregulatory monocytes arise via a single pathway or whether their functional programming is independent of their ontogeny. Indeed, it also remains to be determined whether monocytes with pro-tumor properties are ontogenetically distinct from tumor antigen-presenting monocyte subsets. Similarly, Lyve1^lo^ MHCII^hi^ CX3CR1^hi^ and Lyve1^hi^ MHCII^lo^ CX3CR1^lo^ interstitial macrophages appear to arise separately from distinct monocyte subsets (35), but it is unclear at what stage of differentiation their developmental pathways diverge.

Studies of mice and humans have revealed that fetal, neonatal, and young and old adult monocytes have distinct basal transcriptional profiles and exhibit differential responses to cytokines and microbial stimulation (71–73), but single-cell studies are now required to determine to what degree this variation reflects the monocyte subset composition. Microenvironmental differences in the fetal liver and neonatal and adult bone marrow niches likely impact monopoiesis, but monocyte progenitors and differentiation pathways during fetal development and the neonatal period are less well-defined, as are the effects of aging. The impact of the microbiome is another important area of research. Circulating components derived from commensal organisms, as well as short-chain fatty acids they produce, have been demonstrated to impact myelopoiesis (74–77), but further study is required to define their effects on the production and functional programming of specific monocyte subsets.

Multiparametric single-cell profiling (transcriptomic, proteomic and epigenomic), fate mapping and other technical developments have improved our understanding of monocyte subsets, differentiation and ontogeny in recent years, and combinatorial approaches will continue to advance our knowledge in this field as these tools become more widely accessible. Lineage trajectory analyses using omics datasets, along with fate mapping studies, will permit the distinction between monocyte subsets representing transitional states of the same cells vs. cells derived independently via separate pathways. It will also be critical to pair single-cell omics profiling and functional analyses, with careful interpretation where bulk populations of cells are used for functional studies. Precise identification of monocyte subsets will be facilitated by identification of new surface markers and reporter mice that enable tracking of specific subpopulations e.g., suppressive monocytes within a heterogeneous fraction of monocytic cells.

It will of course be particularly important to evaluate the ontogeny of monocyte subsets in humans as well as mice. Single-cell RNA sequencing datasets have already revealed heterogeneity among mouse and human monocytes (11, 28, 29, 36, 37), and allowed comparison between mouse and human subsets, which informs the extrapolation of observations made in studies of murine monocyte ontogeny to humans.

Ultimately, studies defining mechanisms of monocyte subset production and maintenance of unique monocyte identities have the potential to facilitate the design of therapeutic interventions to target specific monocyte subsets in a variety of disease contexts, including infectious and inflammatory diseases, cancer and aging. It remains to be seen whether developmental targeting will be an effective strategy for clinical use.

## Author Contributions

All authors listed have made a substantial, direct and intellectual contribution to the work, and approved it for publication.

### Conflict of Interest Statement

The authors declare that the research was conducted in the absence of any commercial or financial relationships that could be construed as a potential conflict of interest.
